# A Route to Dicyanomethylene Pyridines and Substituted Benzonitriles Utilizing Malononitrile Dimer as a Precursor

**DOI:** 10.3390/molecules16010298

**Published:** 2011-01-04

**Authors:** Noha M. Helmy, Fatma E. M. El-Baih, Monirah A. Al-Alshaikh, Moustafa S. Moustafa

**Affiliations:** 1 Women Students-Medical Studies & Sciences Sections, Chemistry Department, College of Science, King Saud University, Riyadh, KSA, P.O. Box 22452, Riyadh 11495, Saudi Arabia; 2 Department of Chemistry, Faculty of Science, University of Kuwait, Safat 13060, Kuwait

**Keywords:** malononitrile dimer, arlymethylenemalononitrile, benzylidenemalononitrile, pyrazolopyridine

## Abstract

The conditions of the reaction of malononitrile dimer with enaminones and arylidenemalononitrile could be adapted to yield either pyridines or benzene derivatives. A new synthesis of pyrido[1,2-*a*]pyrimidines from the reaction of malononitrile dimer **1** and 2-phenyl-3-piperidin-1-yl-acrylonitrile (**11)** is described. Compound **1** condensed with DMFDMA to yield an enaminonitrile that reacted with hydrazine hydrate to yield *N*',4,6-triamino-2*H*-pyrazolo[3,4-*b*]pyridine-5-carboxamidine (**17**).

## 1. Introduction

**Scheme 1:**
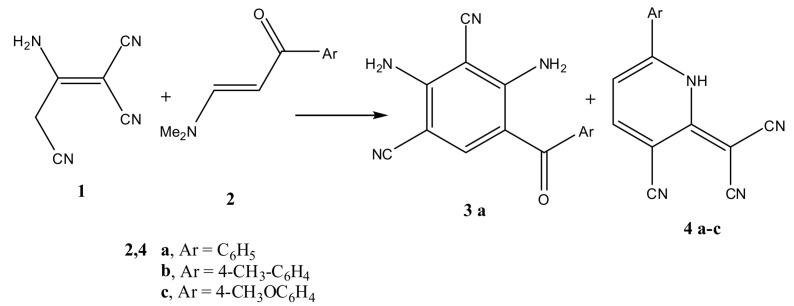
Malononitrile dimer as precursor to heterocycles and substituted benzenes [11].

Polyfunctionally substituted nitriles are versatile reagents that have been extensively utilized in the past as precursors to polyfunctionally substituted heteroaromatics [1,2,3,4]. Interest in further developing the synthetic potential of these compounds has been revived [5,6,7]. 2-Aminoprop-1-ene-1,1,3-tricarbonitrile (**1**) has proved to be an excellent precursor to condensed pyridines, pyridazines, and pyrazoles [8,9,10]. However, to our knowledge the utility of **1** as a precursor to polyfunctional aromatics has received little attention. Elnagdi *et al*. have noted the formation of **3** as a side product from the reaction of **2** with **1**, while compound **4** was obtained as the main product [11] (Scheme 1). In connection to results reported earlier [11] we were able to react **1** with **5a,b** to afford either pyridines or benzene derivatives after changing the reaction conditions. 

## 2. Results and Discussion

**Scheme 2:**
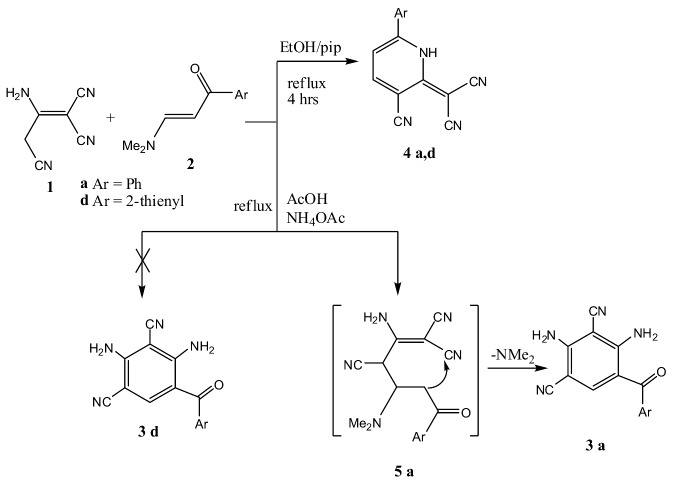
Synthesis of pyridine **3a** and substituted benzenes **4a,d**.

Thus reaction of **1** with **2a,b** in ethanolic piperidine has afforded **4a,d** as a sole product. On the other hand, when the reaction was conducted in acetic acid in the presence of ammonium acetate and refluxing for 4 hrs only **3a** was formed via intermediate **5** (Scheme 2) The ^1^H-NMR of **4a**, in addition to phenyl proton signals, showed two doublets at δ = 7.16 ppm and δ = 7.86 ppm with *J* = 8 Hz, typical for pyridine H-5 and H-4, respectively. A D_2_O exchangeable one proton signal for a NH group appeared at δ = 9.42 ppm. 

The ^1^H-NMR spectrum of **3a** revealed a singlet at δ = 8.29 ppm for H-6 and two D_2_O exchangeable amino signals at δ = 7.08 ppm and δ = 7.77 ppm, in addition to the phenyl protons (see Experimental). The ^13^C-NMR clearly indicated the carbonyl carbon at δ = 190.23 ppm and two CN signals at δ = 114.13 and 113.85 ppm.

In an attempt to generate further examples of the synthesis of substituted benzenes **3**, malononitrile dimer **1** was reacted with **2d** in acetic acid/ammonium acetate, and a product with molecular formula C_13_H_8_N_4_OS (M^+^ at *m/z* = 268) which we think to be **3d** was isolated after reflux for 1/2 hrs; prolonged heating did not change the identity of the compound. The ^1^H-NMR under D_2_O exchange of the presumed structure **3d**; showed, along with three thienyl protons, two amino group singlets at δ = 7.1 ppm and δ = 7.9 ppm, and a doublet at δ = 8.2 ppm with *J* = 8 Hz, that could not be explained or assigned to any proton in the suggested structure. 

**Scheme 3:**
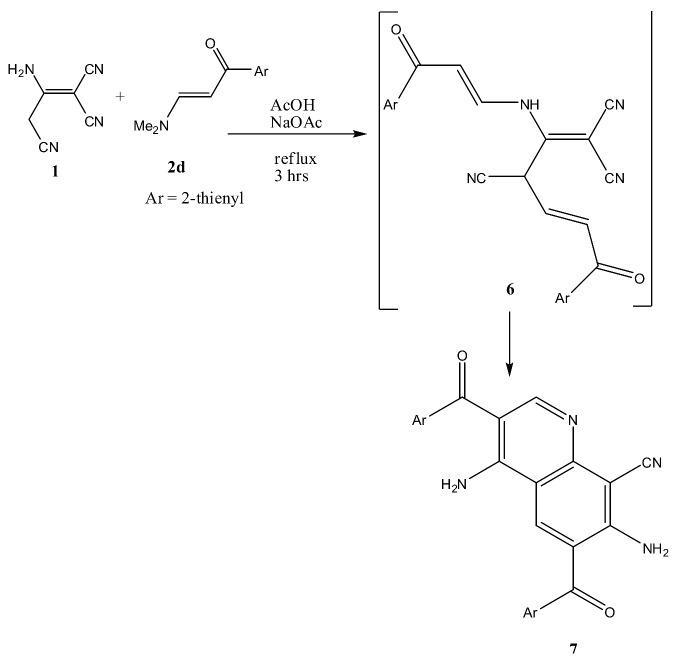
Synthesis of 4,7-diamino-3,6-di(thiophene-2-carbonyl)quinoline-8-carbonitrile (**7**).

Repeating the same reaction using sodium acetate instead of ammonium acetate, and refluxing for 3 hrs, a whole new set of data were obtained. A compound with molecular formula C_20_H_12_N_4_O_2_S_2_ (M^+^ 404) was obtained. ^1^H-NMR of this compound showed two singlets at δ= 7.47 ppm and δ= 8.98 ppm each for one proton of C-5 and C-2 of the pyridine ring, respectively, in addition to six thienyl protons and two amino signals. The ^13^C-NMR spectrum showed the presence of 19 different carbon atoms with two carbonyl carbons at δ = 184.7 ppm. These data can be interpreted as corresponding to structure **7** that is assumed to result from initial reaction of the active methylene moiety and the amino function in **1** with **2d** to yield the intermediate **6** that then cylizes to **7** (Scheme 3). 

**Scheme 4:**
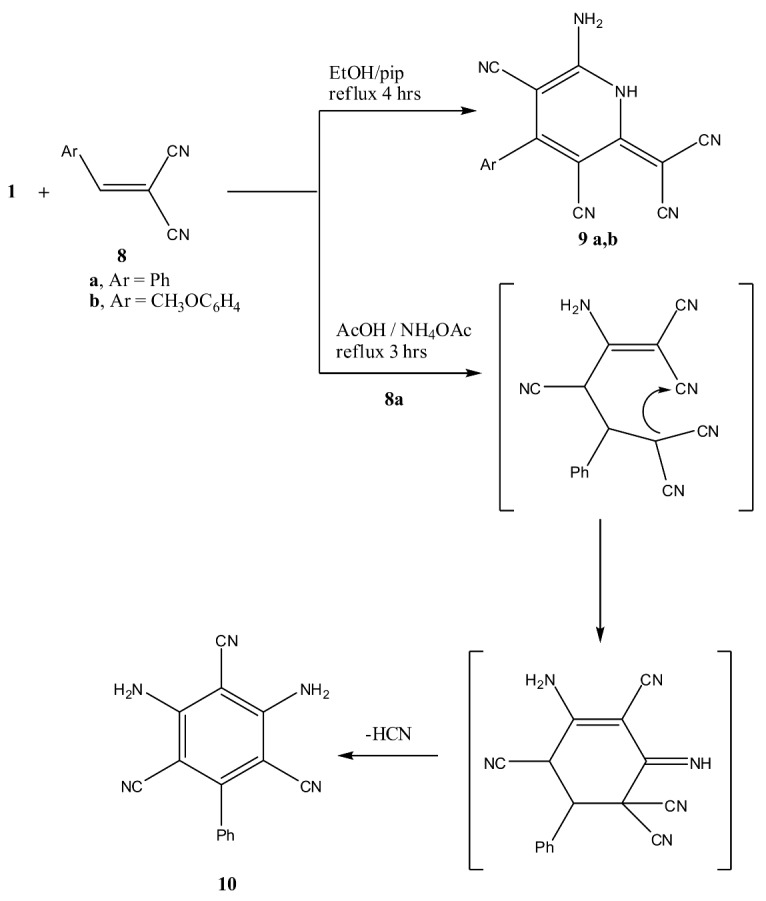
Synthesis of 6-amino-2-dicyanomethylene-4-aryl-2,3-dihydropyridine-3,5-dicarbonitriles **9a,b** and 3,5-diaminobiphenyl-2,4,6-tricarbonitrile (**10**).

Like the recently reported formation of **9** from reaction of **1** and **8a,b** in ethanolic chitosan, compound **1** reacted with **8a,b** to yield dihydropyridine **9a,b**. However in refluxing acetic acid in the presence of ammonium acetate, the benzene derivative **10** was obtained as indicated by the spectral data (Scheme 4).

**Scheme 5:**
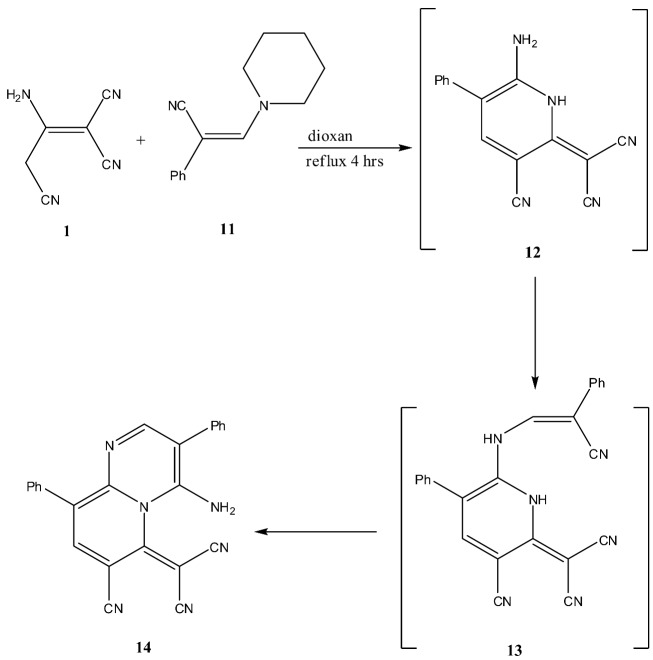
Synthesis of 2-(4-amino-7-cyano-3,9-diphenyl-pyrido[1,2-*a*]pyrimidin-6-ylidene)-malononitrile (**14**).

**Scheme 6:**
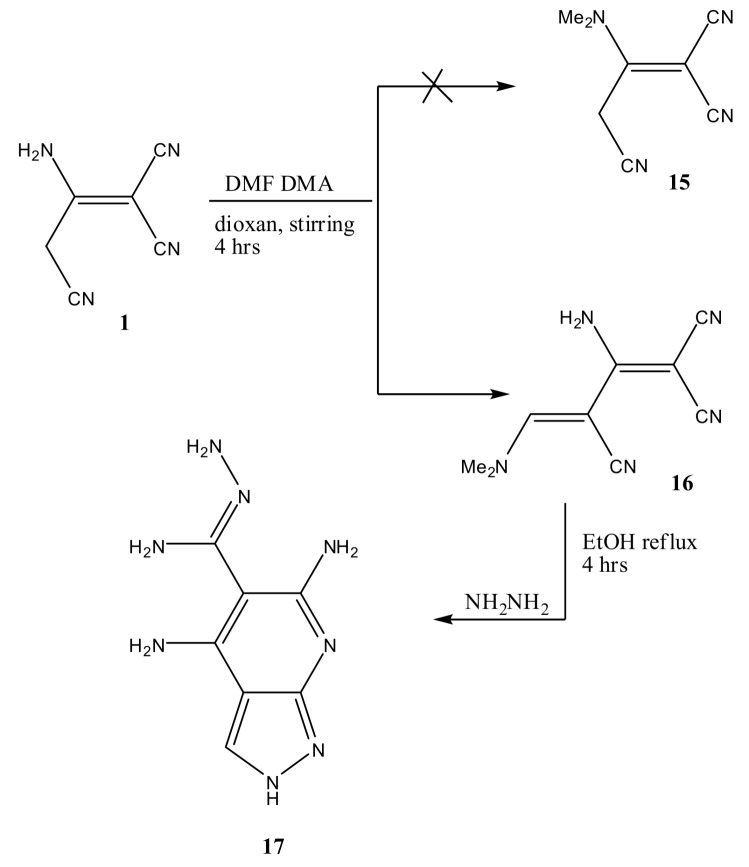
Synthesis of 4,6-diamino-2H-pyrazolo[3,4-b]pyridine-5-carboxamide hydrazone (**17**).

The reaction of **1** with **11**, which was recently obtained by reacting benzyl cyanide with triethyl orthoformate and piperidine [12], afforded **14** via intermediates **12** and **13**. Attempts to isolate **13** have failed (cf. Scheme 5). The reaction of **1** with DMFDMA afforded **16** which may exist in *E* or *Z* forms. Isomeric structure **15** was ruled out based on ^1^H NMR that revealed the D_2_O exchangeable amino signal at δ = 7.19 ppm. In addition, the ^13^C NMR did not reveal any signals for the sp^3^ carbons other than those of the dimethylamino moiety. Reacting **16** with hydrazine hydrate afforded 4,6-diamino-2*H*-pyrazolo[3,4-*b*]pyridine-5-carboxamide hydrazone **17** (cf. Scheme 6). 

## 3. Experimental

### 3.1. General

All melting points are uncorrected and were determined on a Sanyo (Gallenkamp) instrument. Infrared spectra were recorded from KBr discs on a Perkin-Elmer 2000 FT–IR system.^1^H-NMR and ^13^C-NMR spectra were determined on a Bruker DPX spectrometer operating at 400 MHz for ^1^H-NMR and 100 MHz for ^13^C-NMR using DMSO-d_6_ as solvent and TMS as internal standard; chemical shifts are reported in δ (ppm). Mass spectra were measured on VG Autospec Q MS 30 and MS 9 (AEI) spectrometers, with EI at 70 eV'. Elemental analyses were measured by means of LEOCHNS-932 Elemental Analyzer. General purpose silica gel on polyester 20 x 20 cm TLC plates with UV indicator were used in TLC experiments to monitor completion of reactions, in which ethyl acetate-petroleum ether (1:1) was used as eluent.

### 3.2. 2,4-Diamino-5-benzoyl-isophthalonitrile *(**3a**)*

A mixture of 2-aminoprop-1-ene-1,1,3-tricarbonitrile (**1**, 1.32 g, 0.01 mol) and enaminone **2a** (0.01 mol) in AcOH (10 cm) and 0.2 gm of NH_4_OAc, was kept at reflux temperature for 4 hrs. The mixture was cooled and then poured onto ice-water. The solid, so formed, was collected by filtration and recrystallized from EtOH to give yellow crystals; Yield 83%; m.p. 298–299 °C; Anal. Calcd. for C_15_H_10_N_4_O (262.27): C, 68.69; H, 3.84; N, 21.36%. Found: C, 68.53; H, 3.92; N, 21.34%; IR (KBr, cm^−1^): 3,443, 3,352 (NH_2_), 3,322, 3,209 (NH_2_), 2,210, 2,206 (2CN); ^1^H-NMR: δ, ppm = 7.08 (br s, 2H, NH_2_, D_2_O exchangeable) 7.50–7.57 (3H, m, H-3',4',5'), 7.77 (2H, br. s, NH_2_, D_2_O exchangeable), 8.22 (2H, dd, ^3^*J* = 8.0, ^4^*J* = 1.6, H-2',6'), 8.29 (1H, s, H-6); ^13^C-NMR: δ, ppm = 190.23, 159.85, 159.62, 157.90, 154.44, 154.12, 143.65, 136.01, 130.21 (2C), 127.32 (2C), 115.89, 114.13, 113.85; MS: *m/z* (%) 262 (M^+^, 100), 234 (15), 217 (5), 192 (5), 164 (25), 131 (10).

### 3.3. General procedure for the synthesis of compounds ***4a,d***

A mixture of 2-aminoprop-1-ene-1,1,3-tricarbonitrile (**1**, 1.32 g, 0.01 mol) and enaminone **2a,b** (0.01 mol) in EtOH (10 mL) was treated with piperidine (5 drops). The reaction mixture was refluxed for 4 h. The mixture was cooled and then poured onto ice-water. The solid, so formed, was collected by filtration and recrystallized from EtOH to give yellow crystals.

*2-(3-Cyano-6-phenyl-1H-pyridin-2-ylidene)malononitrile* (**4a**). Yield 80%; m.p. 257–259 °C; Anal. Calcd. for C_15_H_8_N_4_ (244.26): C, 73.76; H, 3.30; N, 22.94%. Found: C, 73.94; H, 3.52; N, 23.04%; IR (KBr, cm^−1^): 3,097 (NH), 2,210, 2,182 (3CN); ^1^H-NMR: δ, ppm = 7.16 (1H, d, *J* = 8.0 Hz, H-5), 7.48–7.51 (3H, m, H-3',4',5'), 7.86 (1H, d, *J* = 8.0 Hz, H-4), 8.01 (2H, m, H-2',6'), 9.42 (1H, br. s, NH, D_2_O exchangeable); ^13^C-NMR: δ, ppm = 180.88, 160.54, 159.45, 155.50, 136.89, 132.09, 128.31 (2C), 127.32 (2C), 114.72, 113.88 (2C), 91.82, 63.25; MS: *m/z* (%) 243 (M^+^, 100), 217 (25), 152 (25), 128 (15), 105 (100), 77 (10).

*2-(3-Cyano-6-thiophen-2-yl-1H-pyrid in-2-ylidene)malononitrile* (**4d**). Yield 75%; m.p. 200–202 °C; Anal. Calcd. for C_13_H_6_N_4_S (250.28): C, 62.93; H, 2.42; N, 22.39; S, 12.81%. Found: C, 63.06; H, 2.54; N, 22.54; S, 12.98%; IR (KBr, cm^−1^): 3,189 (NH), 2,230, 2,210 (3CN); ^1^H-NMR: δ, ppm = 7.04 (1H, d, *J* = 8.0, H-5), 7.14 (1H, t, *J* = 4.0, thienyl H-4'), 7.66–7.68 (2H, m, H-4, thienyl H-3'), 7.76 (1H, d, *J* = 4.0, thienyl H-5'), 8.06 (1H, br. s, NH, D_2_O exchangeable); MS: *m/z* (%) 250 (M^+^, 100), 223 (20), 185 (30), 158 (15), 141 (20), 114 (25), 82 (10), 69 (15).

### 3.4. Synthesis of 4,7-diamino-3,6-di(thiophene-2-carbonyl)quinoline-8-carbonitrile *(**7**)*.

A mixture of 2-aminoprop-1-ene-1,1,3-tricarbonitrile (**1**, 1.32 g, 0.01 mol) and enaminone **2d** (0.01 mol) in AcOH (10 cm) and 0.2 gm of NH_4_OAc, was kept at reflux temperature for 3 hrs. The mixture was cooled and then poured onto ice-water. The solid so formed was collected by filtration and recrystallized from EtOH to give yellow crystals; Yield 88%; m.p. 330–332 °C; Anal. Calcd. for C_20_H_12_N_4_O_2_S_2_ (404): C, 59.40; H, 2.97; N, 13.86; O, 7.92; S, 15.84%. Found: C, 59.50; H, 2.78; N, 13.96; O, 7.87; S, 15.89%.; ^1^H-NMR: δ, ppm = 7.05 (br. s, 2H, NH_2_), 7.27 (t, *J* = 4.0, 1H, thienyl H-4'), 7.31 (t, *J* = 4.0, 1H, thienyl H-4''), 7.47 (s, 1H, H-5), 7.79 (d, 1H, *J* = 3.2, thienyl H-3'), 7.85 (d, 3H, *J* = 3.2, thienyl H-3''& NH_2_), 7.93 (d, 1H, *J* = 5.2, H-5'), 8.17 (d, 1H, *J* = 5.2, H-5''), 8.98 (s, 1H, H-2); ^13^C-NMR: δ, ppm = 128.9, 154.0 (2C), 184.7 (2 C=O), 77.1, 98.3, 103.9, 115.85, 118.0, 129.2,131.6, 134.0, 135.7, 136.1, 140.6, 143.67, 156.1, 157.1, 160.4, 162.0; MS: *m/z* (%) 404 (M^+^, 100), 373 (10), 358 (5), 319 (20), 187 (5), 11 (20).

### 3.5. General procedure for the synthesis of compounds ***9a,b***

A mixture of 2-aminoprop-1-ene-1,1,3-tricarbonitrile (**1**, 1.32 g, 0.01 mol) and enaminone **8a,b** (0.01 mol) in EtOH (10 mL) as a solvent was treated with piperidine (5 drops). The reaction mixture was refluxed for 4 hr. The mixture was cooled and then poured onto ice-water. The solid so formed was collected by filtration and recrystallized from EtOH to give yellow crystals.

*6-Amino-2-dicyanomethylene-4-phenyl-2,3-dihydro-pyridine-3,5-dicarbonitrile* (**9a**). Yield 82%; m.p. 195–197 °C; Anal. Calcd. for C_16_H_8_N_6_ (284.28): C, 67.60; H, 2.84; N, 29.56%. Found: C, 67.43; H, 2.61; N, 29.33%; IR (KBr, cm^−1^): 3,467, 3,323 (NH_2_), 3,222 (NH), 2,314, 2,219 (4CN); ^1^H-NMR: δ, ppm = 7.52–7.61 (7H, m, Ar-H, NH_2_, D_2_O exchangeable), 8.19 (1H, br. s, NH, D_2_O exchangeable); ^13^C-NMR: δ, ppm = 160.35, 158.81, 158.71, 133.82, 130.47, 128.76 (2C), 128.67, 128.36 (2C), 116.10, 114.60, 113.5, 95.29, 89.17; MS: *m/z* (%) 284 (M^+^, 100), 257 (25), 219 (10), 165 (25), 127 (10), 77 (5).

*2-[6-Amino-3-aminoethynyl-5-cyano-4-(4-methoxy-phenyl)-1H-pyridin-2-ylidene]malononitrile* (**9b**). Yield 82%; m.p. 248–250 °C; Anal. Calcd. for C_17_H_10_N_6_O (314.31): C, 64.96; H, 3.21; N, 26.74%. Found: C, 65.01; H, 3.22; N, 26.44%; IR (KBr, cm^−1^): 3,423, 3,327 (NH_2_), 3,212 (NH), 2,187, 2,150 (3CN); ^1^H-NMR: δ, ppm = 3.82 (s, 3H, OCH_3_), 6.86 (br, 2H, NH_2_, D_2_O exchangeable), 7.04 (2H, d, *J* = 8.0, H-3',5'), 7.35 (2H, d, *J* = 8.0, H-2',6'), 8.22 (1H, br. s, NH, D_2_O exchangeable); ^13^C-NMR: *δ*, ppm = 162.95, 160.23, 159.47, 158.97, 133.45, 130.19 (2C), 127.80, 121.39, 117.24, 116.92, 113.82 (2C), 85.26, 80.48, 55.31; MS: *m/z* (%) 284 (M^+^, 100), 257 (25), 219 (10), 165 (25), 127 (10), 77 (5).

### 3.6. Synthesis of 3,5-Diaminobiphenyl-2,4,6-tricarbonitrile *(**10**)*

A mixture of 2-aminoprop-1-ene-1,1,3-tricarbonitrile (**1**, 1.32 g, 0.01 mol) and benzylidene-malononitrile (**8**, 0.01 mol)in AcOH (10 cm) and 0.2 gm of NH_4_OAc, was kept under reflux temperature for 3 hr. The mixture was cooled and then poured onto ice-water. The solid so formed was collected by filtration and recrystallized from EtOH to give yellow crystals; yield 80%; m.p. 290–292 °C; Anal. Calcd. for C_15_H_9_N_5_ (259.27): C, 69.49; H, 3.50; N, 27.01%. Found: C, 69.62; H, 3.34; N, 27.17%; IR (KBr, cm^−1^): 3,371, 3,305 (NH_2_), 3,265, 3,213 (NH_2_), 2,218 (3CN); ^1^H NMR: δ, ppm = 4.51 (br. s, 4H, 2NH_2_, D_2_O exchangeable), 7.41–7.44 (2H, m, H-3',5'), 7.50–7.53 (3H, m, H-2',4',6'); ^13^C NMR: δ, ppm = 160.92 (2C), 157.07, 135.08, 129.92, 128.49 (2C), 128.21 (2C), 115.61 (2C), 85.65, 81.06, 75.95, 66.31; MS: *m/z* (%) 259 (M^+^, 100), 234 (20), 205 (15), 165 (20), 127 (10), 77 (50).

### 3.7. Synthesis of 2-(4-Amino-7-cyano-3,9-diphenylpyrido[1,2-a]pyrimidin-6-ylidene)-malononitrile *(**14**)*

A mixture of 2-aminoprop-1-ene-1,1,3-tricarbonitrile (**1**, 1.32 g, 0.01 mol) and 2-phenyl-3-piperidin-1-yl-acrylonitrile (**11**, 0.01 mol) in dioxane (10 mL) was kept under reflux temperature for 3-4 hrs. The mixture was cooled and then poured onto ice-water. The solid so formed was collected by filtration and recrystallized from AcOH to give yellow crystals; yield 85%; m.p. 270–272 °C; Anal. Calcd. for C_24_H_14_N_6_ (386.42): C, 64.60; H, 3.65; N, 21.75%. Found: C, 64.48; H, 3.55; N, 21.90%; IR (KBr, cm^−1^): 3,383, 3,186 (NH_2_), 2,237, 2,196 (3CN); ^1^H-NMR: δ, ppm = 6.82 (2H, br. s, NH_2_, D_2_O exchangeable), 7.24–7.52 (m, 12H, Ar-H); MS: *m/z* (%) 386 (M^+^, 10), 379 (40), 337 (70), 319 (100), 278 (95), 259 (35), 251 (30), 210 (20), 179 (20), 155 (10), 140 (25), 115 (15), 140 (25), 115 (10), 77 (15), 59 (20).

### 3.8. Synthesis of 2-amino-4-(dimethylamino)buta-1,3-diene-1,1,3-tricarbonitrile *(**16**)*

A mixture of 2-aminoprop-1-ene-1,1,3-tricarbonitrile (**1**, 1.32 g, 0.01 mol) and DMFDMA (1.19 g, 0.01 mol) in dioxane (10 mL) was stirred for 3–4 hrs. The mixture then poured onto ice-water. The solid, so formed, was collected by filtration and recrystallized from EtOH to give yellow crystals; yield 90 % m.p. 189–190 °C. *Anal*. Calcd. for C_9_H_9_N_5_ (187.2): C, 57.74; H, 4.85; N, 37.41%. Found: C, 57.61; H, 4.57; N, 37.19%; IR (KBr, cm^−1^): 3,344, 3,221 (NH_2_), 2,208, 2,193 (3CN); ^1^H NMR (400 MHz, DMSO-d_6_): δ, ppm = 2.50 (3H, s, CH_3_), 2.57 (3H, s, CH_3_), 7.19 (br. s, 2H, NH_2_, D_2_O exchangeable), 8.54 (1H, s, olefinic CH); MS: *m/z* (%) 187 (M^+^, 100), 172 (30), 159 (35), 144 (25), 122 (60), 117 (20), 97 (15), 95 (15), 81 (20), 67 (20), 57 (30).

### 3.9. Synthesis of 4,6-diamino-2H-pyrazolo[3,4-b]pyridine-5-carboxamide hydrazone *(**17**)*

A mixture of **13** (1.87 g, 0.01 mol) and hydrazine monohydrate (1.00 g, 0.02 mol) in EtOH (20 mL) was refluxed for 3–4. The mixture then poured onto ice-water. The solid so formed was collected by filtration and recrystallized from EtOH to give a faint yellow product; yield 87 %; m.p. 210–212 °C; Anal. Calcd. for C_7_H_10_N_8_ (206.21): C, 40.77; H, 4.89; N, 54.34%. Found: C, 40.58; H, 4.65; N, 54.05%; IR (KBr, cm^−1^): complicated signals from 3,402 to 3,156 for (NH) and (4NH_2_); ^1^H-NMR: δ, ppm = 5.34 (2H, br. s, NH_2_, D_2_O exchangeable), 5.97 (2H, br. s, NH_2_, D_2_O exchangeable), 7.10 (2H, br. s, NH_2_, D_2_O exchangeable), 7.29 (2H, br. s, NH_2_, D_2_O exchangeable), 8.00 (1H, s, H-3), 8.82 (1H, br. s, NH, D_2_O exchangeable); MS: *m/z* (%) 106 (M^+^, 100), 190 (95), 174 (100), 159 (75), 145 (40), 109 (35), 92 (40), 77 (60), 67 (100).

## 4. Conclusions

We could successfully utilize **1** as precursor to a variety of polyfunctionally substituted aminoaromatics that seem of value as potential precursors to dyes and pharmaceuticals.
